# Evaluation of blood supply and metabolism in tumor, axillary lymph node and normal breast tissue with F-18 FDG PET/CT in breast cancer: comparison with pathological prognostic factors

**DOI:** 10.1186/s12905-023-02858-3

**Published:** 2024-01-16

**Authors:** Ummuhan Abdulrezzak, Hulya Akgun, Ahmet Tutus, Mustafa Kula, Serap Dogan, Abdullah Bahadır Oz, Engin Ok

**Affiliations:** 1 Department of Nuclear Medicine, Erciyes University, School of Medicine, Kayseri, Turkey; 2 Department of Pathology, Erciyes University, School of Medicine, Kayseri, Turkey; 3 Department of Radiology, Erciyes University, School of Medicine, Kayseri, Turkey; 4 Department of General Surgery, Erciyes University, School of Medicine, Kayseri, Turkey

**Keywords:** F-18 FDG PET/CT, Blood flow-glucose metabolism, Breast cancer

## Abstract

**Background and purpose:**

Perfusion parameters obtained in F-18 FDG PET/CT performed for staging purposes in breast cancers may provide additional information about tumor biology as well as glucose metabolism. The aim of this study was to evaluate throughout F-18 FDG PET/CT the relationship between blood flow and glucose metabolism and histological parameters of the primary tumor, normal mammary gland, and axillary lymph nodes in breast cancer patients.

**Materials and methods:**

Sixty six female patients (mean age 51 y ± 12,81) were prospectively included to this study. We performed dynamic blood flow (*f*) study that started with 296–444 MBq (8–12 mCi) F-18 FDG injection and lasted for 10 minutes, and glucose metabolism (*m*) imaging one hour later. On each frame, mean activity concentration (AC) values (Bq/mL) were recorded on a spherical volume of interest (VOI) having a volume of ~ 1 cm3 on the hottest voxel of primary tumor (T), across normal breast gland (NG) and ipsilaterally axillary lymph nodes (iLN). Correlations among PET parameters and estrogen receptor (ER), progesterone receptor (PR), human epidermal growth factor receptor 2 (c-erbB2) and Ki67 index were analyzed.

**Results:**

T volume (TV) ranged from 1.1 to 85.28 cm3 [median (IR): 6.44 (11.78)]. There were positive correlations between c-erbB2 and TAC*f* and between c-erbB2 and iLNAC*f* (*p* = 0.045, r = + 0.248; *p* = 0.050, r = + 0.242). In the ER positive (ERP) patients, TV and TAC*m* were significantly lower than those of ER negative (ERN) (respectively *p* = 0.044 and *p* = 0.041). In patients with two positive Ki-67 indices, iLN-SUVmax was significantly higher than one-positive patients (*p* = 0.020). There was a negative correlation between NGAC*m* and histological grade of tumor (*p* = 0.005, r = − 0.365).

**Conclusions:**

Breast cancer shows differences in progression, metastasis and survival due to its diversity in terms of molecular, biological and angiogenesis. High glucose metabolism in breast cancers is associated with tumor aggressiveness. Being able to examine tumor tissue characteristics such as blood flow and glucose metabolism with a single diagnostic technique and to reveal its relationship with histological parameters can provide a reliable pretherapeutic evaluation in breast cancers.

## Introduction

Although the treatment of breast cancers depends on the biological characteristics and molecular markers of each tumor, such as tumor size, lymph node involvement, distant metastases, histological grade, estrogen receptor (ER), progesterone receptor (PR), and human epidermal growth factor receptor 2 (c-erbB2) status, the response to the appropriate treatment strategy may not always be the same in patient groups with similar criteria, and more different and advanced treatment may be required [1, 2].

On the other hand, another important parameter in tumor treatment is the vascular structure of tumors that is nutrients, oxygen, metabolites and therapeutics that may affect the sensitivity of the tumor to radiation and chemotherapy are transported in this way [3].

In breast cancer, knowledge about the biological profile, angiogenic and metabolic status of the tumor and the relationship between all these prognostic parameters plays a very important role in the management of the disease and in predicting the treatment outcome [4]. However, examining all these parameters with separate tests is both difficult in terms of application, costly and time consuming.

Metabolic imaging with F-18 fluorodeoxyglucose positron emission tomography and computed tomography (F-18 FDG PET/CT) is now routinely used as a diagnostic tool for staging, restaging and evaluation of response to treatment in breast cancers [5, 6]. Today, it is possible to characterize the vascularity and biological properties of tumors with the same imaging method by using dynamic blood flow protocols in addition to standard late scanning protocols in F-18 FDG PET/CT, which gives information about glucose metabolism of tissues.

There are some studies evaluating the blood flow-glucose metabolism of breast cancers with different techniques. For example, while morphological and blood flow kinetic findings of breast cancer were characterized by magnetic resonance imaging (MRI) or computed tomography (CT) in some studies, glucose metabolism was analyzed with F-18 FDG PET [7, 8]. Others have evaluated blood flow and metabolism parameters using different radiopharmaceuticals such as H2-O15 and F-18 FDG, which reveal perfusion and glucose metabolism separately [9, 10].

Subsequently, several studies have been presented that simultaneously measure both tumor blood flow and metabolism in F-18 FDG PET/CT [11, 12].

In recent years, there have been publications on the tumor microenvironment, which consists of normal cells, inflammatory cells, molecules and blood vessels that surround and nourish a tumor cell, formed by tumor cells and tumor-induced interactions, as well as tumor tissue in cancer research. And there is evidence that this tumor microenvironment can influence how a tumor grows and spreads [13, 14]. As the functions and characteristics of these inflammatory and other cell groups infiltrating tumor localization and their local interactions with other cells are understood, the role of the microenvironment in shaping cellular events in health and disease has come to be appreciated [15, 16].

Based on this, we hypothesized that some pathways may be activated not only in the tumor tissue and the microenvironment adjacent to the tumor, but also in normal tissues during the cancer development process in patients. And with PET/CT, the molecular imaging method we currently have, we aimed to reveal not only tumor morphology and function, but also possible changes in the perfusion and metabolism of the tumor and normal tissues. As far as we know, a perfusion-metabolism study of the breast that included primary tumor (T), across normal breast gland (NG), and ipsilateral axillary lymph nodes (iLN) even considering MRI, CT, and PET/CT data has not been done in cancer patients. Our aim in this study is to evaluate the changes in blood flow and glucose metabolism of T, NG and iLN by dynamic and static analysis of F-18 FDG PET/CT in breast cancer patients, and to examine the relationship between PET parameters and pathological markers.

## Materials and methods

### Patients

Sixty six female patients (mean age, 51 y; range, 25–80 y) with histologically confirmed breast carcinoma were prospectively included to this study. Inclusion criteria for the study were the presence of biopsy-proven breast carcinoma, no start any treatment and no pregnancy. The institutional review board gave permission in accordance with the ethical principles of the Declaration of Helsinki for the study. Informed consent was obtained from all patients with regard to dynamic and static F-18 FDG PET/CT examination. Patients with locally advanced breast cancer without distant organ metastases on PET and other imaging were selected as candidates for systemic neoadjuvant chemotherapy and the others underwent either breast-conserving surgery or mastectomy.

### Histopathologic analysis

Histological grading was applied by using the Nottingham Histologic Score system (the Elston-Ellis modification of Scarff-Bloom-Richardson grading system) based on tubule formation, nuclear features-pleomorphism and mitotic activity in such a way as to be from 1 to 3 (10) [17]. Each score was then added to give a final total score ranging from 3 to 9. Tumor histological grade was defined according to the final score as following; Grade 1: Tumors with a score of 3–5. Grade 2: Tumors with a score of 6–7. Grade 3: Tumors with a score of 8–9.

Immunostaining of the paraffin-embedded tissue sections were analysed according to Allred score for ER and PR. The positivity of c-erbB2 oncoprotein was determined by HerceptTestTM DAKO test and was scored as 0 (absent), 1 (weak), 2 (moderate), and 3 (strong). Following this assessment if breast cancer negative for estrogen receptor (ERN), progesterone receptor (PRN), and c-erbB2 (c-erbB2N), defined as triple negative (TrN).

Ki67 levels (MIB-1; Immunotech, Westbrook, ME) reflecting cellular proliferation were assessed by calculating the ratio of expression of positive cells per field to total cells per field and results: < 14%, negative (Ki67N) and weakly positive; ≥ 14%: recorded as positive and strongly positive (Ki67P). A cut-off point of 14% was used to distinguish between the categories of low and high proliferative tumors.

### F-18 FDG PET/CT imaging protocol

FDG PET/CT images were performed with a dedicated whole body PET scanner (Gemini TF PET/CT scanner, Philips Medical Systems, Cleveland, Ohio, USA) equipped with a high-resolution PET scanner and a 16-slice Briliance CT scanner. Patients were instructed to fast for at least 6 hours prior to tracer injection. An intravenous cannula was placed inside a vein in the arm contralateral to the tumor side in order to avoid artificial tracer retention in ipsilateral axilla and blood glucose was measured in drawn sample to ensure glucose blood levels below 180 mg/dL. Patients were placed in the supine position on the PET/CT device bed and approximately 296–444 MBq (8–12 mCi) of F-18 FDG followed by 10 mL of saline solution was injected IV via cannula. With the IV FDG application, dynamic *f*-shooting was started for 10 minutes (10 frames of 1 minute) from the chest region, including the patient’s axillary regions.

The patient was then taken to the waiting room and rested, and a static PET/CT scan was performed for *m* imaging 50–60 minutes after the dynamic acquisition. Initially, low-dose CT (120 kVp, 80 mAs, transaxial FOV 600 mm, Pitch: 1.1, slice thickness: 5 mm) without contrast agent was taken from the base of the skull to the mid-thigh or the whole body for use in attenuation correction. Subsequently, a high resolution supine PET dataset (axial full-width at half-maximum 8 mm) over the same region was obtained in 5–8 bed positions with an acquisition time of 1 min for each bed position. PET images were reconstructed using iterative reconstruction with CT-derived attenuation correction using the ordered subsets expectation maximization algorithm. PET, CT and fused PET/CT images were reviewed in multiplanar reconstructions using the software provided by the manufacturer (Extended Brilliance Workstation, Philips Medical System).

### Image analysis

**Fig. 1:**
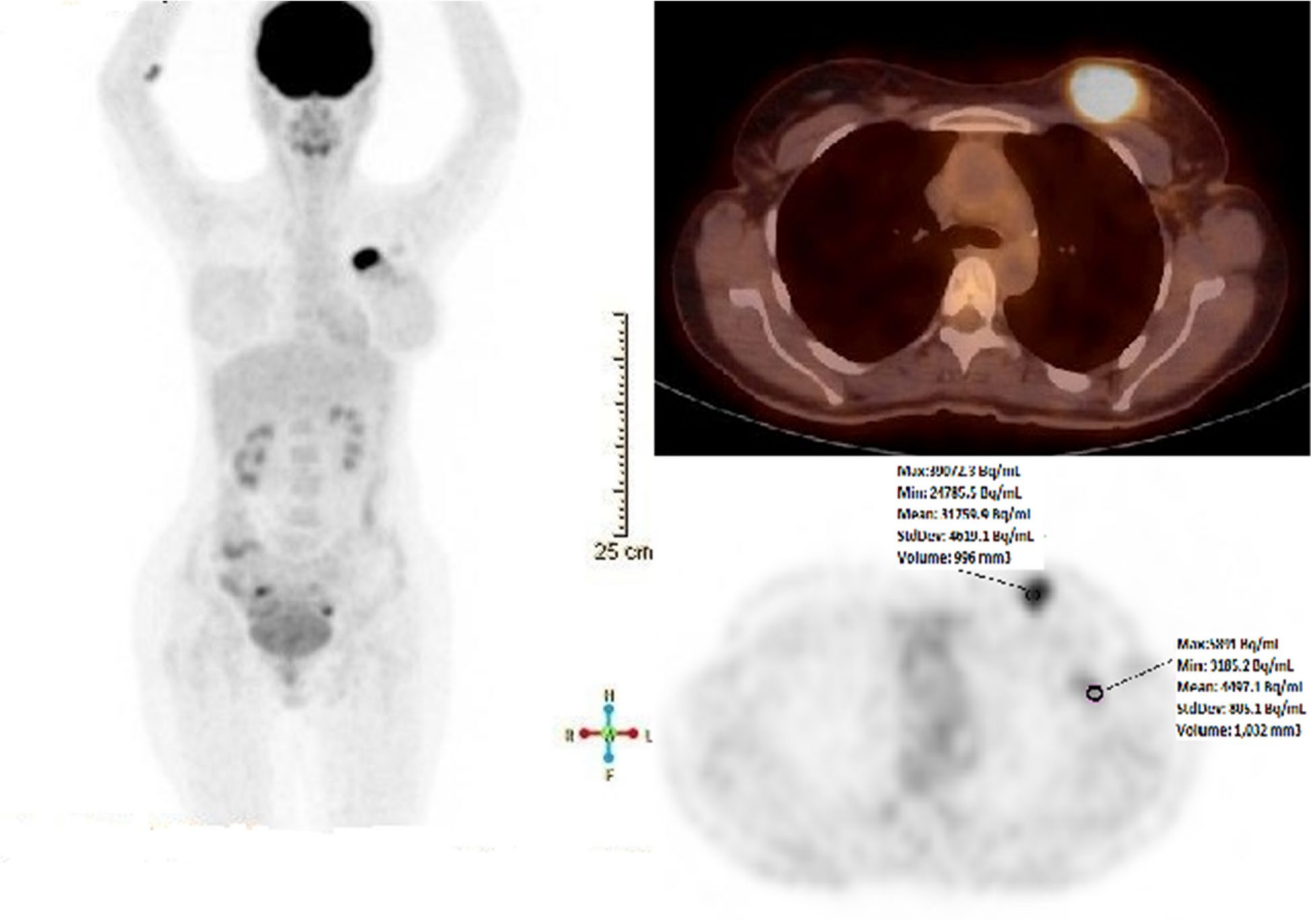
MIP (maximum Intensity Projection) image and transaxial fusion and transaxial PET images from the breast lodge of a 41-year-old female patient

Average activity concentration (AC) values (Bq/mL) were recorded by taking a spherical volume of interest (VOI) with a volume of ~ 1 cm3 in the hottest voxel on T, NG and iLN in blood supply and metabolism images (Fig. 1).

**Fig. 2:**
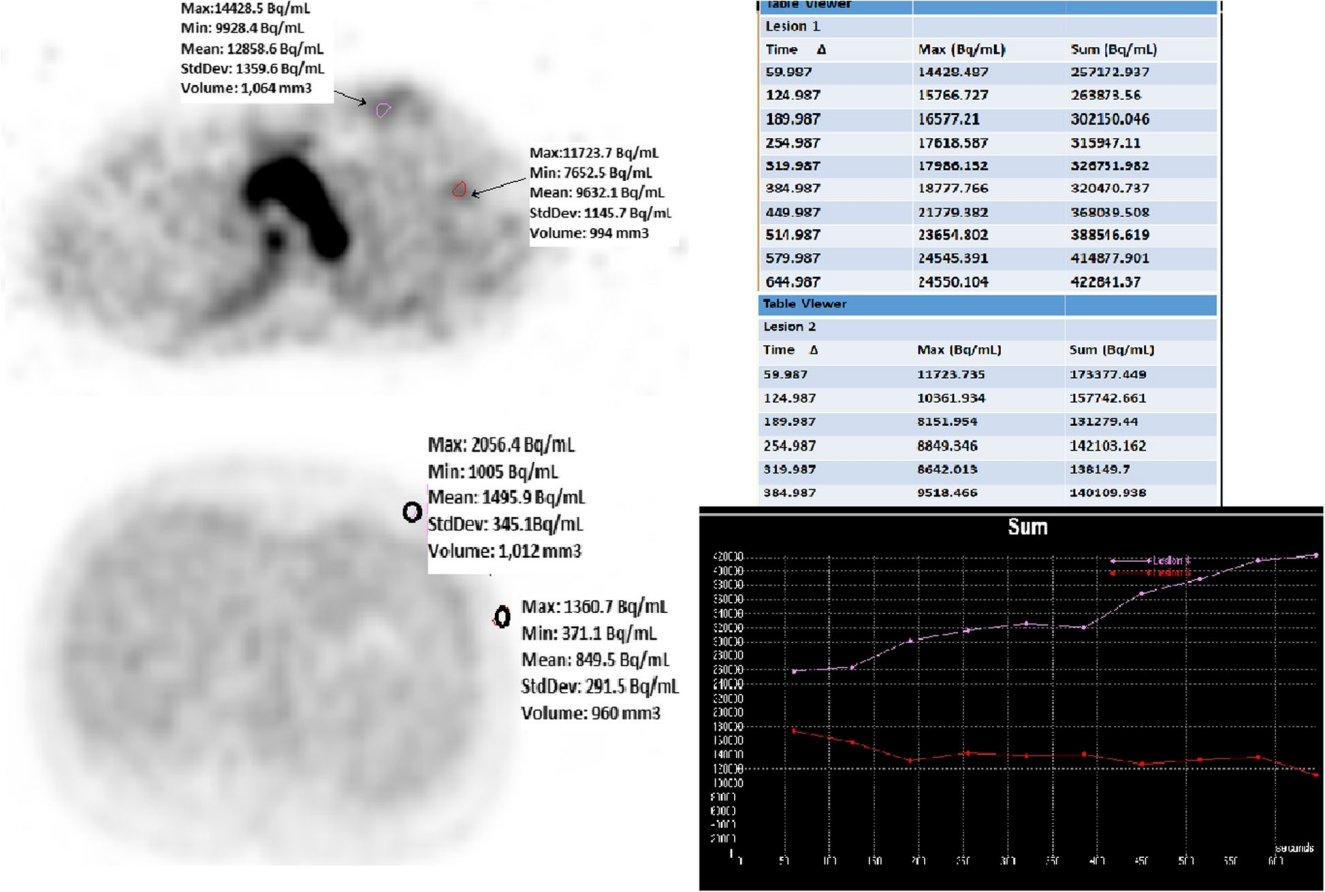
Activity concentration per volume measurements of a 41-year-old female patient with a left breast tumor by taking a volumetric area of interest of approximately 1 cm3 from the breast tumor and ipsilateral lymph node in the dynamic blood flow and late static metabolism transaxial PET images

Tumor volumes (TV) were calculated in cm3, with areas of interest drawn around the tumor, including adjacent voxels with 35% of the SUVmax value of the voxel from which the hottest count was obtained from the *m* images. Maximum standard uptake (SUVmax) values were calculated from the highest pixel of T, NG and iLN in *m* images (Fig. 2).

### Statistical analysis

Statistical analysis included descriptive data for all tested parameters. For all measurements, median ± interquartile range (IR) was calculated for dynamic and static PET parameters and histopathologic parameters. Spearman correlation coefficients were calculated to measure strength of association between tumor dynamic quantitative parameters (median AC values, SUVmax values and histopathologic markers). Associations between the *f* patterns, *m* and histological parameters were evaluated using the bivariate correlation test and non-parametric tests. A *p* value < 0.05 was considered statistically significant.

## Results

The patient demographic data and tumor characteristics have been described in Table 1 and are summarized here. The mean patient age was 51 y ± 12,81 (range 25–80 y).

**Table 1:**
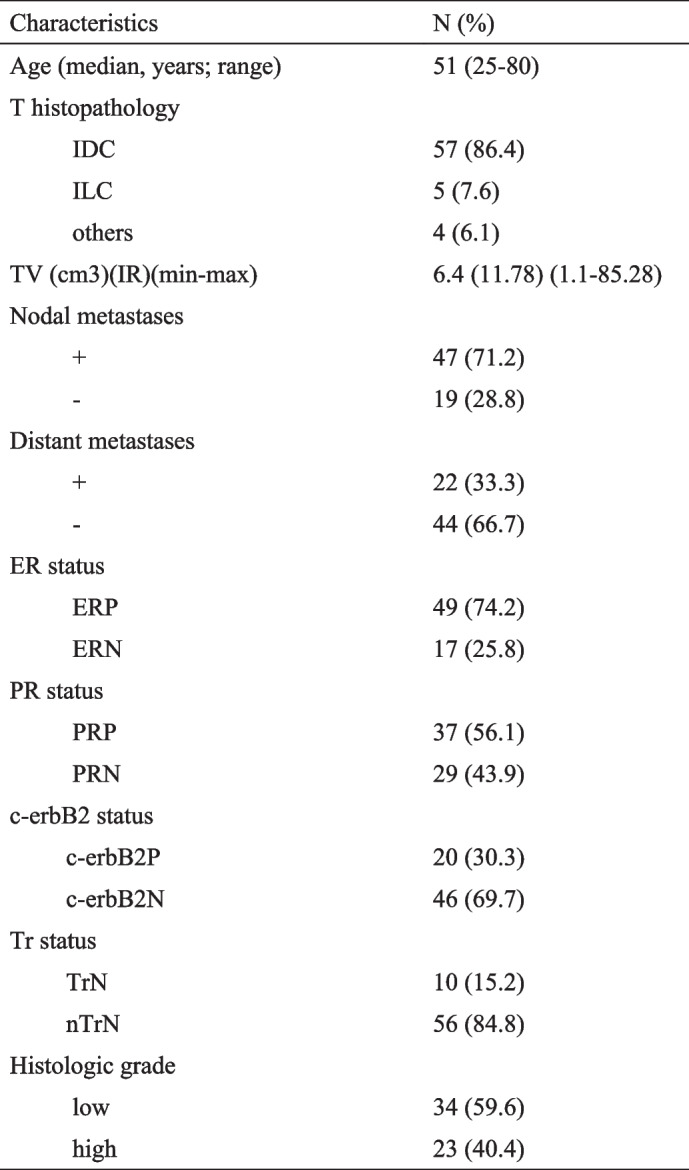
The patient demographic data and tumor characteristics

*T *tumor, *IDC *invasive ductal carcinoma, *ILC* invasive lobular carcinoma, *TV* tumor volume, *IR *interquartile range, *ER *estrogen receptor, *ERP* estrogen receptor positive, *ERN* estrogen receptor negative, *PR* progesterone receptore, *PRP* progesterone receptore positive, *PRN* progesterone receptore negative, *c-erbB2* epidermal growth factor receptor, *c-erbB2P* epidermal growth factor receptor positive, *c-erbB2N* epidermal growth factor receptor negative, *Tr* Ttiple, *TrN* Triple negative, *nTrN* non Triple negative

### Histopathologic results

The mean age of 66 patients included in the study was 51 (age range: 25–80) and TV range was 1.1–85.28 cm3 [median (IR): 6.44 (11.78)]. Other parameters such as tumor histopathology and hormonal status, presence of nodal and distant metastases are given in Table 1. Tubul formation status of the tumors were grade 1 in 2 cases (3.5%), grade 2 in 15 cases (26.3%) and grade 3 in 40 cases (70.2%). Nuclear pleomorphism status of the tumors were grade 1 in 1 case (1.8%), grade 2 in 33 cases (57.9%) and grade 3 in 23 cases (40.4%). Mitotic activity status of the tumors were grade 1 in 23 cases (40.4%), grade 2 in 24 cases (42.1%) and grade 3 in 10 cases (17.5%). Histologic grading of the tumors was determined as grade 1 in 9 cases (15.8%), grade 2 in 25 cases (43.9%) and grade 3 in 23 cases (40.4%). Grade 1 and grade 2 subjects were recorded as low grade (34, %59.6), whereas grade 3 subjects were recorded as high grade (23, %40.4).

## 18F-FDG PET/CT parameters

### Blood flow (*f*) parameters

Two separate timed scans were performed on a total of 66 patients (dynamic first-pass *f* imaging that starts with the injection and lasts for 10 minutes, and late static *m* imaging 50–60 minutes after the injection). Comparisons of *f* parameters between groups of ERN/ERP, PRN/PRP, c-erbB2 negative (c-erbB2N)/c-erb positive (c-erbB2P), triple negative (TrN)/non-triple negative (nTrN) and Ki67N/Ki67P were performed using Mann Whitney U test. AC*f* values of T, NG and iLN in ERP and PRP cases showed lower values compared to ERN and PRN cases. Interestingly, among these, decrease in NG-AC*f* values in ERP cases was statistically significant compared to ERN cases (*p* = 0.049). Unlike the ER and PR results, c-erbB2P subjects showed high AC*f* values in T, NG and iLN, although statistically insignificant compared to c-erbB2N subjects.

Next, we assessed the correlations between blood flow parameters of T, NG and iLN and clinicopathological parameters (Table 2).

**Table 2:**
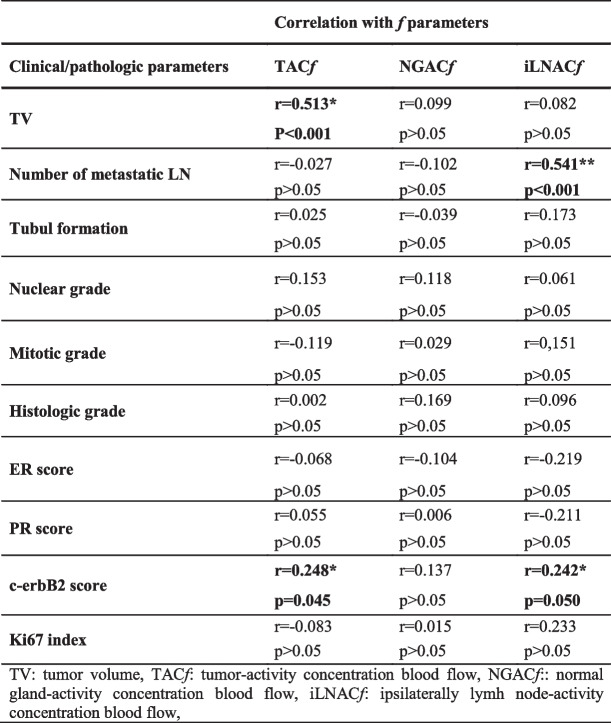
Correlation of blood flow (*f*) parameters and clinicopathologic parameters of breast tumor (*n* = 66)

There was a significant correlation of tumor volume with the TAC*f* (*p* < 0.001, *r* = 0.513). There was also a positive correlation between c-erbB2 and TAC*f* (*p* = 0.045, *r* = + 0.248) and between c-erbB2 and iLNAC*f* (*p* = 0.050, *r* = + 0.242).

### Glucose metabolism (*m*) parameters

Histopathological parameters and blood flow-glucose metabolism parameters were compared in the patients (Table 3).

**Table 3:** Comparison of the blood flow-glucose metabolism parameters in patients with Estrogen Receptor /Progesterone Receptor/ c-erbB2 negative-positive, Triple negative-Non-triple negative and Ki67 1+ and 2+ breast cancer

		glucose metabolism (*m*) parameters of F-18 FDG PET/CT						
Histologic parameters		TV median (IR)	T-SUVmax median (IR)	TAC*m* median (IR)	NG-SUVmax median (IR)	NGAC*m* median (IR)	iLN-SUVmax median (IR)	iLNAC*m* median (IR)
**ER**	**+**	5,76 (7,72)	7,7 (9,7)	9932 (9559)	1,5 (0,55)	1632 (1423)	3,7 (6,9)	4210 (9311)
	**–**	9,84 (24,12)	6,5 (6,85)	14,960 (15257)	1,4 (0,73)	1474 (2014)	5,6 (15,55)	5883 (21220)
	***p***	0,044*	> 0,05	0,041*	> 0,05	> 0,05	> 0,05	> 0,05
**PR**	**+**	4,80 (6,75)	5,9 (6,9)	8932 (9708)	1,5 (0,55)	1520 (1358)	3,7 (6,35)	4210 (5828)
	**–**	8,88 (1783)	7,2 (7,5)	13,033 (14223)	1,4 (0,78)	1475 (1883)	4,7 (12,0)	5856 (18383)
	***p***	0,019*	> 0,05	> 0,05	0,043*	> 0,05	> 0,05	> 0,05
**c-erbB2**	**+**	6,96 (17,96)	6,8 (7,93)	10,547 (8806)	1,6 (0,68)	1709 (1547)	3,95 (12,93)	5671 (20145)
	**–**	6,44 (7,46)	6,85 (7,13)	12,030 (9323)	1,5 (0,6)	1475 (1743)	3,6 (7,38)	3770 (9865)
	***p***	> 0,05	> 0,05	> 0,05	> 0,05	> 0,05	> 0,05	> 0,05
**Tr**	**TrN**	12,8 (41,34)	9,8 (14,4)	20,629 (12580)	1,0 (0,75)	1475 (1818)	5,15 (10,4)	5637 (17207)
	**nTrN**	6,24 (7,86)	6,45 (6,73)	10,137 (8944)	1,5 (0,50)	1576 (1700)	3,7 (7,98)	4422 (11021)
	***p***	> 0,05	> 0,05	0,011*	> 0,05	> 0,05	> 0,05	> 0,05
**Ki-67**	**1+**	6,08 (7,36)	6,1 (5,7)	11,998 (10098)	1,7 (0,7)	1431 (1748)	2,4 (2,4)	2608 (3832)
	**2+**	6,64 (12,4)	6,9 (7,4)	10,752 (9336)	1,5 (0,70)	1520 (1837)	5,6 (10,4)	5485 (16927)
	***p***	> 0,05	> 0,05	> 0,05	> 0,05	> 0,05	0,02*	> 0,05
**Histologic grade**	**low**	6.08 (10.74)	5.70 (5,13)	112,030 (9608)	1.50 (0.70)	2061 (1631)	3.60 (9.50)	4422 (14145)
	**high**	6.52 (8.06)	8,95 (7,15)	12,450 (10716)	1.50 (0.65)	1181 (1376)	4.20 (8.33)	5252 (8887)
	***p***	> 0,05	0.048*	> 0,05	> 0,05	0,003*	> 0,05	> 0,05

*TV* tumor volume, *ER *estrogen receptor, *PR *progesterone receptor, *c-erbB2 *human epidermal growth factor receptor 2, *Tr* Triple, *TrN* triple negative, *nTrN* non triple negative, *Ki-67 1+* <%14, negative and weakly positive, *Ki-67 2+ *≥ %14, strongly positive, *m* metabolism, *T* tumor, *NG* normal breast gland, *iLN* ipsilaterally lymh node, *ACm *activity concentration metabolism, *SUVmax* maximum standardized uptake value, *IR *interquartile range

TV was lower in ERP and PRP patients than in ERN and PRN (*p* = 0.044 and *p* = 0.019, respectively). TAC*m* was lower in ERP patients than in ERN patients (p = 0.041). Similar to the NGAC*f* value, the NG-SUVmax value of the PRP patients was also higher than the PRN patients (*p* = 0.043). In TrN patients, TAC*m* value was found significantly higher than nTrN patients (*p* = 0.011). The cases with Ki-67P showed the higher iLN-SUVmax value according to Ki-67 N cases (*p* = 0.02).

NG-SUVmax values of the iLNP patients [median (IR): 1,4 (0,62)] were lower than those of iLNN patients [median (IR): 1,7 (0,4)] (*p* = 0.017).

The Spearman’s rank correlation analysis was also performed between clinicopathological parameters and *m* parameters of T, NG and iLN (Table 4).

**Table 4:**
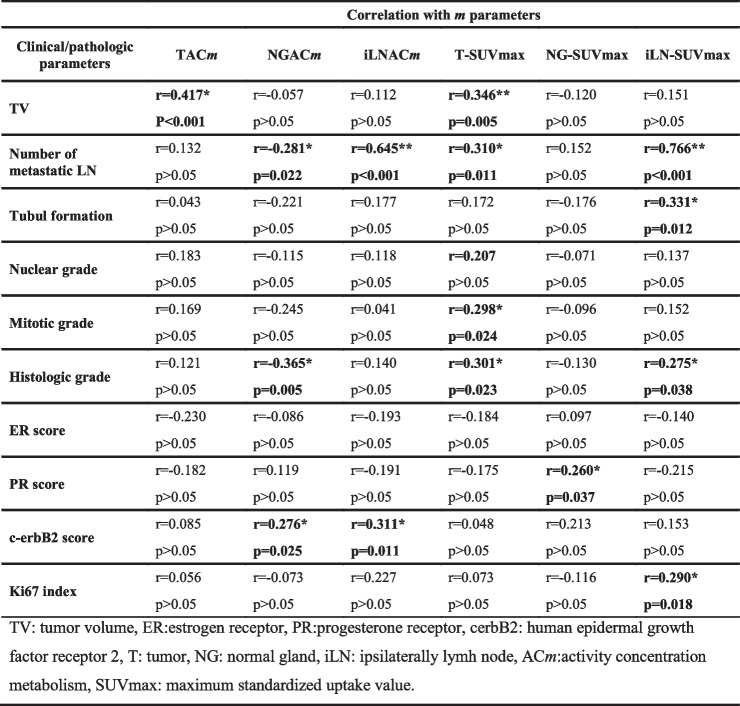
Bivariate Analysis of Correlation Between Blood Flow –Glucose Metabolism Parameters and Measures and Clinicopathologic Parameters of Breast Tumor

Although both TAC*m* and T-SUVmax were positively correlated with TV (*p* < 0.001, r = 0.417 and *p* = 0.005, r = 0.346), there was no significant correlation between TV and *m* parameters of NG and iLN. T-SUVmax value showed positive correlations with mitotic and histologic grade of the tumor (respectively *p* = 0.024, r = 0.298 and *p* = 0.023, r = 0.301), while iLN-SUVmax value showed positive correlations with tubul formation, histologic grade and Ki-67 index (*p* = 0.012, *r* = 0.331; *p* = 0.038, *r* = 275 and *p* = 0.018, *r* = 290). No significant correlations were found between nuclear grade of T and *m* parameters of T (TAC*m*, T-SUVmax), NG (NGAC*m*, NG-SUVmax) and iLN (iLNAC*m*, iLN-SUVmax). Interestingly, there was a moderate negative correlation between the histologic grade of the tumor and NGAC*m* (*p* = 0.005, *r* = − 0.365).

When we looked at the correlations between *m* parameters and ER, PR or c-erbB2 status of T, we saw that ER and PR scores showed negative correlations with all *m* parameters of T (TAC*m*, T-SUVmax) and iLN (iLNAC*m* and iLN-SUVmax), however, these correlations were not statistically significant (*p* > 0.05). Unlike the T and iLN, PR score was significantly positive correlated with NG-SUVmax value (*p* = 0.037, *r* = 0.260). Similarly, c-erbB2 score was significantly positive correlated with both NGAC*m* and iLNAC*m* (*p* = 0.025, *r* = 0.276 and *p* = 0.011, *r* = 0.311).

## Discussion

Although aerobic glycolysis is recognized as an important distinguishing metabolic feature of cancer, uncertainties exist regarding cancer progression and the relationship between perfusion and glycolysis [18, 19]. While there is a close relationship between blood flow, which provides the transport and use of energy substrates, and energy metabolism in normal tissues, this ratio is impaired in tumoral tissues. Although tumor growth and distant metastases are related to increased vascularization, it is advocated by many studies that aggressive tumors exhibit increased glucose metabolism and decreased blood flow (glucose-blood flow mismatch) [7–10]. An advantage of functional imaging modalities such as PET/CT is that dynamic phase images can be obtained after radioactive material application, allowing monitoring of changes in perfusion and time-dependent FDG uptake of lesions. Evaluation of the relationship between in vivo blood supply and metabolism parameters, which can be measured with FDG tracer, and hormonal and other prognostic markers of breast tumors may increase the value of dynamic PET/CT imaging in daily practice. In this prospective study, we aimed to demonstrate the diagnostic utility and feasibility of simultaneous evaluation of blood flow and glucose metabolism in F-18 FDG PET/CT in breast cancer patients while examining the relationship between clinical and histopathological parameters and PET/CT parameters. It is a known fact that increased tumor glucose metabolism in cancer patients is associated with the worse course of the disease. In addition, several previously published studies have suggested that increased metabolic activity and decreased perfusion (higher metabolism/blood flow ratio) predict poor survival [20, 21]. In addition, the absence of ER and PR expression in breast cancers in studies conducted to date indicates a poor prognosis in general, while c-erbB2 and Ki-67 (cell proliferation marker) positivity have been presented as markers showing the invasiveness and metastasis capacity of the tumor [22–24]. In addition to these histopathological markers, it has been shown that microvessel density and tumor angiogenesis in tumors are associated with tumor growth and metastasis [25, 26]. However, as far as we know, the relationship between perfusion and metabolism between tumoral tissue, axillary lymph nodes and normal mammary gland has not been mentioned so far. In our study, in which we examined the relationship between these tissues based on the perfusion and metabolism changes in F-18 FDG PET/CT, we found that NG perfusion was lower in ERP cases than in ERN cases (*p* = 0.049). Consistent with this result, the ER score was negatively correlated with T, iLN, and NG perfusion. In other words, as the estrogen receptor level of the tumor decreased, its blood supply increased. Likewise, as the PR score of the tumor decreased, iLN and NG blood flow increased.

On the other hand, a significant positive correlation was found between c-erbB2 score and T and iLN perfusion. In addition, although not statistically significant, NG perfusion values were found to be higher in c-erbB2 positive cases than in c-erbB2 negative cases. It has been shown in previous publications that cerbB2 positivity is increased in ER and PR negative patients and this marker is associated with advanced clinical stage and histological grade [27]. Yang et al. reported that cerb-B2 positivity was higher in ERN and PRN patients [28]. Our results also support the same findings as the studies mentioned above, but provide some additional information. Apparently, c-erbB2 positivity is a biomarker closely related to microvessel density and perfusion, as well as a marker of tumor aggressiveness in breast cancers. c-erbB2 is an analogue of the rat neu gene found in rat neuroblastomas in the 1980s, localized on the 17q21 chromosome in humans, and acts as a growth factor receptor with tyrosine kinase activity [29]. While c-erbB2 contributes positively to the maintenance of cell growth and transformation states in normal situations, its overexpression in some tumors, such as breast tumors, has been shown to lead to the opposite of this complex process [30]. Microvascularization, which is an important component of cell growth, is a dynamic and multi-step event that takes place between the static and active processes of physiological and pathological conditions. Angiogenesis is as important in cell growth as it is in tumor growth, invasion, and progression. Demonstrating the transition to an angiogenetic phenotype in tumors may guide the determination of tumor subtypes and treatment planning. Vamesu reported that microvessel density was positively correlated with c-erbB2 expression in patients with breast cancer [31]. Slaughter et al. developed the theory of “regional cancer” and Aran D et al. also put forward the theory of “pan-cancer mechanism” based on differential gene expression and protein-protein interaction analysis. In these theories, they reported that pro-inflammatory signals from the tumor stimulate the inflammatory response and that some molecular changes occur in the areas adjacent to the surgically removed tumor, even though there are no tumor cells histopathologically [32, 33]. Apparently, some tumor-induced changes in perfusion and metabolism are possible, not only in the tumor and adjacent areas, but possibly in other normal tissues as well.

As the histological grade of the tumor increased in the patients, the tumor SUVmax value and iLN SUVmax value increased as expected. In patients with high tumor histological grade, glucose metabolism values of the normal mammary gland were significantly lower. Again, glucose metabolism values of the normal mammary gland were low in patients with a high number of metastatic lymph nodes. Although this inverse correlation raises the question of whether there is a relative decrease in the metabolic activity of normal tissue due to increased tumor aggressiveness and metabolism, an interesting finding has puzzled us. As the c-erbB2 score increased in the tumor, the normal mammary gland glucose metabolism (NGACm) value also increased. We have already mentioned above that c-erbB2 positively correlates with perfusion. In c-erbB2 high-expressing tumors, the metabolic activity of both the tumor and lymph nodes and the normal mammary gland were also increased.

Simultaneous measurement of changes in tumor blood flow and glucose metabolism with F-18 FDG PET/CT will provide additional prognostic information by presenting the relationship between perfusion and in vivo tumor biology. Until now, there are few FDG PET/CT studies on metabolic activity and perfusion in breast cancers. There are also publications showing varying degrees of sensitivity and specificity of FDG PET in the detection of metastatic axillary lymph nodes. Our study is an analysis of perfusion and metabolic changes in both primary breast tumor, normal mammary gland and axillary lymph nodes.

The SUVmax of the ipsilateral lymph nodes increased significantly in patients with high Ki-67 level, which indicates tumor proliferation index, compared to patients with low Ki-67 level (*p* = 0.02). Can Ki-67 level predict the tumor’s capacity to metastasize to the lymph node? Martins et al. could not find a relationship between the Ki-67 index of metastatic lymph nodes and surveillance in colorectal cancers, but they stated that there was a positive relationship between the Ki-67 index of the primary tumor and the presence of lymph node metastasis [34]. In our study, we similarly could not find a significant relationship between the Ki-67 index of the primary tumor and other histopathological parameters or perfusion and metabolism parameters. However, there was only a significant positive correlation between Ki-67 index and SUVmax of ipsilateral axillary lymph nodes.

The stroma in the breast cancer microenvironment contains cancer-related fibroblasts, adipocytes, cancer stem cells and immune cells. There is a metabolic interaction between cancer cells and these stromal structures, thanks to different glycolysis-related enzymes and glucose transporter proteins that play an important role in tumor growth, progression, angiogenesis and metastasis [35, 36]. For example, breast cancer-associated fibroblasts have been shown to have an increase in genes related to glycolysis and glucose transporter, and these cells express higher levels of glucose receptors than normal fibroblasts [37] It is also suggested that in breast cancers, unlike other tumors, there is a metabolic interaction through lipid transfer between tumor cells and cancer-related adipocytes, which are stromal cells in the breast microenvironment [38]. In addition to metabolic markers, angiogenesis, which is normally under tight control with the balance between proangiogenic and antiangiogenic factors, is also disrupted in cancer [39]. It has been shown in many different studies that increased glucose metabolism and microvessel density in breast cancers, as in other cancers, are associated with treatment resistance and decreased survival [19–21, 40]. Our study also has some limitations. In order to perform a better statistical analysis, average activity concentration (AC) values (Bq/mL) were calculated in 1 cm3 volume of interest taken from tissues in blood supply and metabolism images. However, in PET/CT images, until now, classical analysis methods have generally been performed with parameters such as SUV, metabolic tumor volume, and total lesion glycolytic activity. Therefore, different studies using these measurement parameters are needed to understand the importance of activity concentration values in unit tissue. In our study, we analyzed perfusion and metabolism parameters in the primary breast tumor and ipsilateral lymph nodes, as well as in the opposite normal breast tissue. However, the change of these parameters in different tissues such as liver, bone tissue and brain, which are distant from the primary tumor and where breast tumors often prefer to metastasize, and evaluating their relationships with histopathological parameters will contribute to the confirmation and enrichment of these data.

## Conclusion

As a result, PET, using FDG, a glucose analogue, is a functional imaging method that uses high amounts of glucose through increased glucose receptors of tumor cells due to the Warburg effect. In this method, it is an extra advantage to take images of the blood supply of the tissues and to evaluate the blood supply parameters quantitatively. We tried to reveal both T, iLN and NG perfusion-metabolism changes in breast cancer patients by performing dynamic and late static studies in FDG PET/CT imaging, unlike the studies done so far. Our results show that patients’ tumor and normal tissue perfusion and metabolism show a number of different relationships with histopathological parameters. Quantitative perfusion parameters in PET/CT can serve as physiological markers of angiogenesis and provide important information about vascular nutrition of tumoral and normal tissues. In addition, simultaneous measurement of changes in tumor blood flow and glucose metabolism with F-18 FDG PET/CT may be a method that demonstrates the relationship between perfusion and in vivo tumor biology in a practical approach. Differences in treatment response between different histological subtypes can be better understood through blood supply and metabolism studies in specific breast cancer subgroups with high patient numbers. In addition, further studies with functional imaging in which the above-mentioned metabolic interactions are more detailed are needed to plan the use of metabolic and perfusion markers as treatment targets in breast cancers.

## Data Availability

The data that support the findings of this study are available on request from the corresponding author. The data are not publicly available due to privacy or ethical restrictions.

## Abbreviations

*f*: blood flow
*m*: glucose metabolism
AC: mean activity concentration
VOI: volume of interest
T: primary tumor
NG: across normal breast gland
iLN: ipsilaterally axillary lymph nodes
TV: tumor volume
ER: estrogen receptor
PR: progesterone receptor
c-erbB2: human epidermal growth factor receptor 2
TAC*f*: primary tumor activity concentration in blood flow images
TAC*m*: primary tumor activity concentration in glucose metabolism images
SUVmax: maximum standardized uptake value
iLNAC*f*: ipsilaterally axillary lymph nodes-activity concentration in blood flow images
iLNAC*m*: ipsilaterally axillary lymph node-activity concentration in glucose metabolism images
NGAC*f*: across normal breast gland activity concentration in blood flow images
NGAC*m*: across normal breast gland activity concentration in glucose metabolism images
ERP: estrogen receptor positive
ERN: estrogen receptor negative
PRP: progesterone receptor positive
PRN: progesterone receptor negative
IR: interquartile range
Ki67P: Ki67 positive
Ki67N: Ki67 negative
TrN: Triple negative
nTrN: Non-Triple negative
